# Arginine Vasopressin Effects on Subjective Judgments and Neural Responses to Same and Other-Sex Faces in Men and Women

**DOI:** 10.3389/fendo.2017.00200

**Published:** 2017-08-21

**Authors:** James K. Rilling, Ting Li, Xiangchuan Chen, Pritam Gautam, Ebrahim Haroon, Richmond R. Thompson

**Affiliations:** ^1^Anthropology, Emory University, Atlanta, GA, United States; ^2^Psychiatry and Behavioral Sciences, Emory University, Atlanta, GA, United States; ^3^Center for Translational and Social Neuroscience, Emory University, Atlanta, GA, United States; ^4^Center for Behavioral Neuroscience, Atlanta, GA, United States; ^5^Psychology, Bowdoin College, Brunswick, ME, United States

**Keywords:** vasopressin, fMRI, face processing, sex differences, nucleus accumbens

## Abstract

Arginine vasopressin (AVP) influences social and emotional behaviors across a wide range of species. In humans, intranasal AVP has been previously shown to alter physiological responses to and subjective judgments of same-sex faces in both men and women. The present study attempted to elucidate the neural mechanism for these effects by randomizing 40 healthy men and 40 healthy women to treatment with either 40 IU intranasal AVP or a saline placebo approximately 30 min before imaging their brain function with fMRI as they viewed same and other-sex faces. All subjects were also scanned a second time several days later with no treatment to evaluate the persistence of AVP effects over time. AVP acutely increased positive ratings of same-sex faces in women, with some evidence that these effects persisted until the second scan. While AVP had no acute effects on same-sex ratings in men, AVP increased positive ratings of same-sex faces several days later. On the other hand, AVP had no effect on other-sex face judgments in either sex. AVP modulation of brain function was focused on the nucleus accumbens (NAc) and the lateral septum, two reward processing areas involved in the formation of social bonds. AVP provoked acute increases in right NAc and bilateral lateral septum responses to female faces among men, with left lateral septum responses persisting over time while right NAc responses reversed over time. Finally, AVP modulated hypothalamic activation to faces in both men and women. The present study therefore indicates that intranasal AVP affects subjective ratings and neural responses to same and other-sex faces in men and women, with some effects persisting and others emerging over time. Future studies should investigate whether AVP effects are modulated by individual variables such as genotype, personality, or attachment style as previously reported for other nonapeptides.

## Introduction

Arginine vasopressin (AVP) is a nine amino acid peptide that is synthesized in the hypothalamus and released into the general circulation where it acts as a hormone to regulate blood pressure and water retention in the body (1, 2). In addition, AVP-producing neurons within and outside the hypothalamus release AVP into the brain where it can act *via* vasopressin receptors (and possibly related oxytocin receptors) to influence social and emotional behaviors.

Arginine vasopressin and arginine vasotocin (AVT, its non-mammalian homolog) effects on brain functions related to social behavior have been explored in numerous non-human animals. These studies have identified both species-specific neuromodulatory circuits that have evolved in relation to unique life histories, as well as circuits that are highly conserved across species. Within these circuits, target areas have been identified in which AVT/AVP can stimulate courtship [hindbrain; rough-skin newt; (3)], promote affiliation related to pair bonding [septum and ventral pallidum, prairie voles; (4, 5)], and increase gregariousness [septum, zebra finches; (6)]. Areas in which the peptides influence aggression and aggressive communication [anterior hypothalamus, hamsters and prairie voles (7–9); amygdala, rats (10); septum, finches and sparrows (11, 12); preoptic area, plainfin midshipmen (13)], as well as social withdrawal [amygdala, rats (14); hindbrain, goldfish (15)] have also been identified. From this body of work, it has become clear that these peptides can influence a variety of social responses, promoting affiliative interactions in some species or contexts and aggressive or antisocial responses in others, *via* actions in different brain circuits [further discussed in Ref. (16)].

Arginine vasopressin effects on human social cognition and behavior have been studied using intranasal AVP administration, which is believed to cross the blood–brain barrier (17). Most studies have been done in men, where intranasal AVP has been found to facilitate cooperation (18, 19), enhance recognition of sexual cues (20), and enhance encoding of happy and angry faces (21). In addition, among both men and women, intranasal AVP increases empathic concern in those who received high levels of paternal warmth (22), as well as anxiety and skin conductance responses to angry faces (23). However, AVP can also have different effects in men and women for some social responses. In men, AVP induced agonistic facial motor patterns and decreased perception of friendliness to faces of unfamiliar men. On the other hand, in women, AVP induced affiliative facial motor patterns and increased perceptions of friendliness in faces of unfamiliar women (23). Together, these studies suggest that, in humans, AVP has sex- and perhaps context-dependent influences on a variety of social responses, as it also does in other vertebrates.

Although we know less about where within the human brain AVP acts to influence particular social responses than we do in other animals, fMRI studies have identified some regions in which AVP alters patterns of activity in parallel with its effects on social behavior and/or emotional processes. In men, intranasal AVP increases the amygdala response to emotional scenes (24), consistent with increased self-reported anxiety (23). To the contrary, it decreased both amygdala and anterior insula activation in response to negative social interactions between men in the iterated Prisoner’s Dilemma (PD) Game (25). On the other hand, intranasal AVP augmented bilateral insula activation to positive social interactions in men the PD game while having the opposite effect in women (26). Thus, AVP may increase the salience of positive social interactions, while decreasing the salience of negative social interactions among men in some contexts. AVP has also been shown to decrease activation within the temporoparietal junction, a key node of the theory of mind network, when viewing unfamiliar but not familiar faces (27). Finally, AVP has been shown to modulate putative emotion regulation circuitry in the human brain. For example, AVP increased connectivity of the right amygdala with the medial prefrontal cortex (mPFC) during the processing of socially threatening scenes, which was interpreted as reflecting a reduced suppressive effect of mPFC on amygdala activity (24). In another study, it induced a relative increase in activation within the subgenual ACC, another key emotion regulation area (28), in response to emotional faces.

To further explore the neural mechanisms underlying AVP’s effects on responses to social stimuli, the present study investigates whether intranasal AVP modulates the BOLD fMRI response to viewing unfamiliar same and other-sex faces in both men and women. Given known effects of AVP on learning (21, 29, 30) and long-lasting, organizational effects on social responsiveness that it and related nonapeptides can have during development (31–34), we also investigated whether any observed effects persist beyond the period of AVP exposure. Although our previous work focused on a lower dose (20 IU), preliminary data from a parallel study suggested that a higher dose (40 IU) more effectively stimulates positive assessments potentially related to affiliative responses to faces in women and may also promote positive responses in men, whereas lower doses promote negative responses (under review, this issue). Our strongest prediction was, therefore, that 40 IU AVP would increase positive responses toward the faces of other women in women. Although lower doses promote negative responses toward same-sex faces in men (23), these preliminary data also led us to predict that this higher dose (40 IU) would promote positive responses in men. We also predicted that if AVP has effects in humans that are, as in other species such as prairie voles and finches, dependent on social contexts, including the sex of the individual/stimulus with which subjects interact (35, 36), then AVP may produce different effects toward same- and other-sex stimuli, In particular, if AVP promotes affiliative responses related to pair bonding in human males (37), then we predicted that any positive effects in males would be selective for female faces. Further, we predicted that positive responses would be concurrent with increased activation in the ventral striatum, in which nonapeptides, including AVP, have been shown to promote affiliative responses in prairie voles, particularly in relation to pair bonding (see above), and in which activity is generally associated with positive social responses in humans (38, 39). In contrast, we predicted any antisocial effects might be concurrent with increased activation in regions in which AVP/AVT act to enhance aggression/antisocial behaviors in other animals, most notably the amygdala and hypothalamus (see references in second paragraph). Predictions about the lateral septum were more difficult; AVT can promote aggression *via* actions there, but also gregariousness, and AVP in the lateral septum promotes affiliative processes related to pair bonding. Further, based on previous findings in humans (28), we predicted that AVP would increase the subgenual ACC response to viewing faces.

## Materials and Methods

### Subjects

Participants were 40 healthy men and 40 healthy women between the ages of 21 and 30 (mean = 23.89 ± SD = 2.19). All participants were heterosexual and not in a committed relationship. Participants were randomized to either 40 IU intranasal AVP (20 men and 20 women) on scan 1 followed by no treatment on scan 2, or to placebo (PL) (20 men and 20 women) on scan 1 followed by no treatment at scan 2. Scans were collected within 2 weeks of each other. The mean interval between scans was 4.3 days (SD = 2.25 days). Randomization was performed by the Emory Investigational Drug Service (IDS) using Research Randomizer,^1^ which randomizes each subject by using the method of randomly permuted blocks.

All potential subjects completed a full medical history questionnaire. Subjects with a history of seizures or other neurological disorders, alcoholism, or any other substance abuse, hypertension, cardiovascular disease, diabetes, and other endocrine diseases or malignancy were excluded from the study. Subjects who reported a history of asthma or migraine headaches were excluded if their symptoms were persistent, disabling, and required one or more medication adjustments within the past month. Subjects with a history of head trauma, psychiatric illness, or use of medications with known psychoactive effects over the past year were generally excluded. However, a *post hoc*, secondary review of screening forms revealed inclusion of one subject who reported mild head trauma and another who indicated seizure due to fever at the age of 2. Subjects with claustrophobia were excluded at the discretion of the Principal Investigator. Subjects were allowed to continue on their current medications if the agents in question were not reported to alter brain activity in regions of interest. Some of these medications included birth control and antihistamines for allergy.

All subjects gave written informed consent, and the study was approved by the Emory University Institutional Review Board and the U.S. Food and Drug Administration. Preparation of study medication and details of randomization were maintained by the Emory IDS and all study personnel including the PI were blind to group assignment. Administration of 40 IU vasopressin was generally safe. None of the subjects developed any major side effects of study medication, including anaphylaxis. One subject experienced a transient increase in blood pressure; however, this subject was in the PL group.

### Preparation and Administration of AVP and Placebo

#### Intranasal AVP

Lyophilized AVP purchased from Polypeptide Group (Hillerod, Denmark) was diluted in sterile saline at concentration of 40 U/0.5 ml. The solution was immediately sterilized *via* a 0.22 μm filter before being transferred to sterile conical tube and stored at −80°C until use. On the day of the study, the drug was transferred to a nasal spray bottle after thawing from which the subjects self administered. Both prior to and after freezing, three AVP samples were tested for sterility and potency by Eagle Analytics. Samples measured 103, 112, and 90.2% of 40 IU, respectively.

#### Intranasal Placebo

The PL group self-administered 0.5 ml of PL spray comprised of sterile saline, pH adjusted and filtered in a similar manner as above, but not containing the neuropeptide, prepared ahead of time and stored at −80°C until use.

#### Administration of AVP or PL

Both experimenters and subjects were blind to the treatment subjects received. All solutions were administered intranasally. The AVP group self-administered 40 IU of AVP (Polypeptide Group, Limhamn, Sweden). This required five nasal puffs to administer 0.5 ml of solution. The PL group self-administered five nasal puffs of PL. Subjects were instructed to place the nasal applicator in one nostril and depress the lever until they felt a mist of spray in the nostril, to then breathe in deeply through the nose, and afterward to place the applicator in the other nostril and repeat the process.

### Monitoring Vital Signs

To monitor for unintended side-effects of AVP administration, subjects’ ear temperature, heart rate, and blood pressure were measured prior to drug administration and again approximately 20 min later.

Following intranasal administration of AVP, CSF concentrations begin rising within 10 min, continue to increase for up to 80 min, and remain above those of PL-treated subjects at 100–120 min after administration (17). Thompson et al. (23) tested subjects at 15 and 50 min after intranasal vasopressin administration. Accordingly, our goal was for subjects to be fully immersed in the task at 50 min post-drug administration. We, therefore, aimed to start both the task and fMRI scan at 30 min after drug administration. In actuality, this time period averaged 31.27 min (SD = 3.82) across subjects.

### Task

Nine head-shot photographs were taken from each of three Caucasian male and three Caucasian female models, displaying neutral expressions. All photos were similar, but unique in terms of dress and lighting.

For scan 1, subjects viewed pictures of two male models, two female models, and one object (a coffee mug). Three pictures of each model and object were displayed per run and there were a total of three runs. We repeated presentations of the same models to increase familiarity with the stimuli during the test to, at least in part, mimic the repeated contact with particular individuals that would typically occur during a social interaction.

Thus, subjects viewed a total of 45 pictures. A single trial involved an 8 s presentation of the stimulus, a variable fixation interval (2, 4, or 6 s) in which subjects viewed a cross in the center of the screen, a 4 s interval during which subjects were asked to rate the stimulus on approachability on a scale from −3 (threatening and unapproachable) to 3 (friendly and approachable), a 0.5 s fixation interval, another 4 s interval during which subjects rated the stimulus on attractiveness on a scale from −3 (unattractive) to 3 (attractive), and finally another variable fixation interval (2, 4, or 6 s). Pictures were presented in pseudorandom order. Total task duration was approximately 21 min.

Scan 2 stimuli were the same as for scan 1, except that we included nine pictures from one additional male model and nine pictures from one additional female model to assess whether any persisting effects of AVP treatment were specific to faces that were seen previously.

E-prime software (Psychology Software Tools, Pittsburgh) was used for stimulus presentation. Stimuli were projected onto a screen that subjects could view through a mirror mounted on the head coil in the MRI scanner. Subject responses were recorded using a response box.

### Neuroimaging Data Acquisition

#### Anatomical Image Acquisition

Subjects lay motionless in a supine position in the scanner with padded head restraint to minimize head movement during scanning. Each scanning session began with a 15 s scout, followed by a 5 min T1-weighted MPRAGE scan (TR = 2,600 ms, TE = 3.02 ms, matrix = 256 × 256, FOV = 256 mm, slice thickness = 1.00 mm, gap = 0 mm).

#### fMRI Image Acquisition

Functional scans used an EPI sequence with the following parameters: TR = 2,000 ms, TE = 28 ms, matrix = 64 × 64, FOV = 224 mm, slice thickness = 2.5 mm, 34 axial slices. TE was minimally decreased from the typical value (32 ms) in order to reduce magnetic susceptibility artifact in the orbitofrontal region. The duration of each EPI scan was about 7 min (15 pictures × 8 s per picture, plus 8 s for fixation, and 8 s to rate each picture on two different adjectives).

### Analysis of Subjective Ratings

Two sample *t*-tests were used to test for between-subject effects of AVP treatment (vs. PL) on approachability and attractiveness ratings of same and other-sex faces at scan 1 to test for acute effects of the drug, and again at scan 2 to test for more prolonged effects, whether acute effects were present or not.

### Analysis of Neuroimaging Data

The analysis was conducted with the Oxford Center for Functional Magnetic Resonance Imaging of the Brain’s software library (FSL).^2^

The preprocessing pipeline of the fMRI data involves (1) motion correction using the MCFLIRT (40), (2) non-brain tissue removal using the BET (41), (3) slice timing correction, (4) high-pass temporal filtering with a cut-off of 200 s, (5) spatially smoothing with a Gaussian kernel of full-width at half maximum (FWHM) of 4 mm, and (6) normalizing to MNI space *via* corresponding extracted T1 brain using Boundary-Based-Registration (42).

For each subject, the preprocessed fMRI data were analyzed using a general linear model (GLM). Regressors were specified for male faces, female faces, and objects seen at both scans, and also for the novel male and female faces seen at scan 2. Each task regressor was convolved with a standardized model of the hemodynamic response function. Contrasts of beta values for male faces vs. objects and female faces vs. objects were generated for use in group analyses. The individual-level GLM was implemented using FMRIB’s Improved Linear Model (FILM).

Given widespread evidence for sex differences in the AVP system (43, 44), analyses were conducted separately for males and females. For group analyses, a two sample *t*-test was used to compare the contrast (face-object) for same and other-sex faces between the AVP and PL groups at scan 1. Another two-sample *t*-test was used to compare the same contrasts between the AVP and PL groups at scan 2 (i.e., carryover effects). Whole brain exploratory analyses were thresholded using clusters determined by *z* > 3.1 (voxel-wise 1-tailed *p* < 0.001), and a family wise error (FWE)-corrected cluster significance threshold of *p* < 0.05 was applied to the suprathreshold clusters. Region of interest (ROI) analyses were also conducted within bilateral nucleus accumbens (NAc), amygdala, lateral septum, and hypothalamus. NAc and amygdala were defined using the Harvard-Oxford Subcortical Structural Atlas implemented in FSL^3^ with 50% probability as a threshold. The lateral septum and hypothalamus were manually defined based on the coordinates and anatomy of these ROIs and surrounding brain structures, referring to the “Atlas of the Human Brain” (45). All ROIs were defined in the MNI space (Figure S1 in Supplementary Material). Results of ROI analyses were corrected for multiple comparisons at the voxel level (*p* < 0.05) using Gaussian Random Field Theory. In addition, we imposed a minimum spatial extent threshold of three voxels. Results for each contrast were also Bonferroni corrected for the number of ROIs investigated (8), such that significance required *p* < 0.006.

Neuroimaging data from one female subject in the placebo group was unusable due to technical problems. Subjects were compensated with a total of $50 on each of the two visits.

## Results

### Participant Demographics in the AVP and PL Groups

There was no difference in age between participants randomized to AVP vs. PL for either men [AVP group: mean = 23.05, SD = 1.99; PL group: mean = 23.50, SD = 2.46; *t*(38) = −0.64, *p* = 0.53] or women [AVP group: mean = 23.40, SD = 1.98; PL group: mean = 23.45, SD = 2.31; *t*(38) = −0.07, *p* = 0.94]. The racial distribution across groups was follows: female AVP = 5 Caucasian, 7 African American, 7 Asian, 1 mixed race; female PL = 8 Caucasian, 5 African American, 6 Asian, 1 Hispanic; Male AVP = 5 Caucasian, 3 African American, 11 Asian, 1 Hispanic; Male PL = 10 Caucasian, 3 African American, 5 Asian, 1 Mixed, 1 not available/other.

### Attractiveness and Approachability Ratings

#### Female Participants

**Figure 1:**
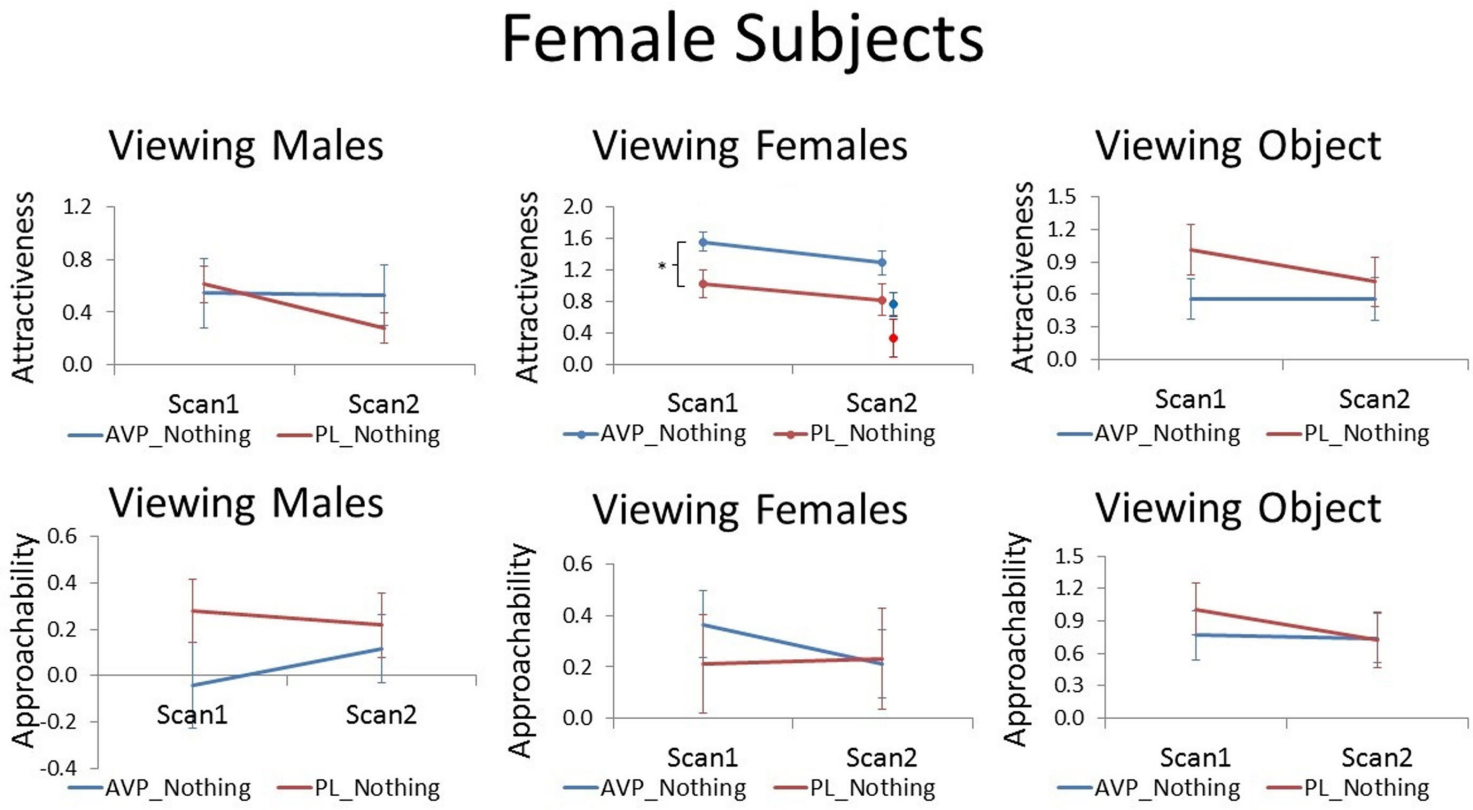
Subjective ratings of same-sex faces, other-sex faces, and objects in women, as a function of arginine vasopressin (AVP) vs. PL treatment and first vs. second scan. For attractiveness ratings of female faces, data for novel faces at scan 2 are plotted to the right of familiar faces. Error bars = ± 1 SE. *p < 0.05 for AVP vs. PL at scan 1.

Arginine vasopressin treatment increased female participant’s attractiveness ratings of female faces compared with PL treatment at scan 1 [*t*(38) = 2.51, *p* = 0.017]. There was a trend for this effect to persist until scan 2 when no treatment was given, although this result was only marginal [*t*(38) = 1.83, *p* = 0.075]. In the PL group, the number of days between scan 1 and scan 2 was not correlated with scan 2 attractiveness ratings of familiar female faces (*r* = −0.03, *p* = −0.91). However, in the AVP group, there was a significant positive correlation (*r* = 0.55, *p* = 0.01) such that female faces were rated as more attractive with increasing scan interval. Thus, AVP effects appear to become more pronounced with longer scan interval (Figure S2 in Supplementary Material). To determine if these marginal carryover effects generalized to the novel faces seen at scan 2, we also compared attractiveness ratings for novel female faces between the AVP and PL groups and found no significant difference [*t*(38) = 1.46, *p* = 0.15]. However, the effect of AVP did not significantly differ for familiar and novel faces [*F*(1, 38) = 0.05, *p* = 0.83] (Figure 1). Approachability ratings for female faces did not differ between the AVP and PL group on either scan 1 or scan 2. Nor was there any significant effect of AVP treatment on either attractiveness or approachability ratings of male faces, at either scan 1 or scan 2. Finally, there was no significant effect of AVP treatment on either attractiveness or approachability ratings of objects, at either scan 1 or scan 2 (Table S1 in Supplementary Material).

#### Male Participants

**Figure 2:**
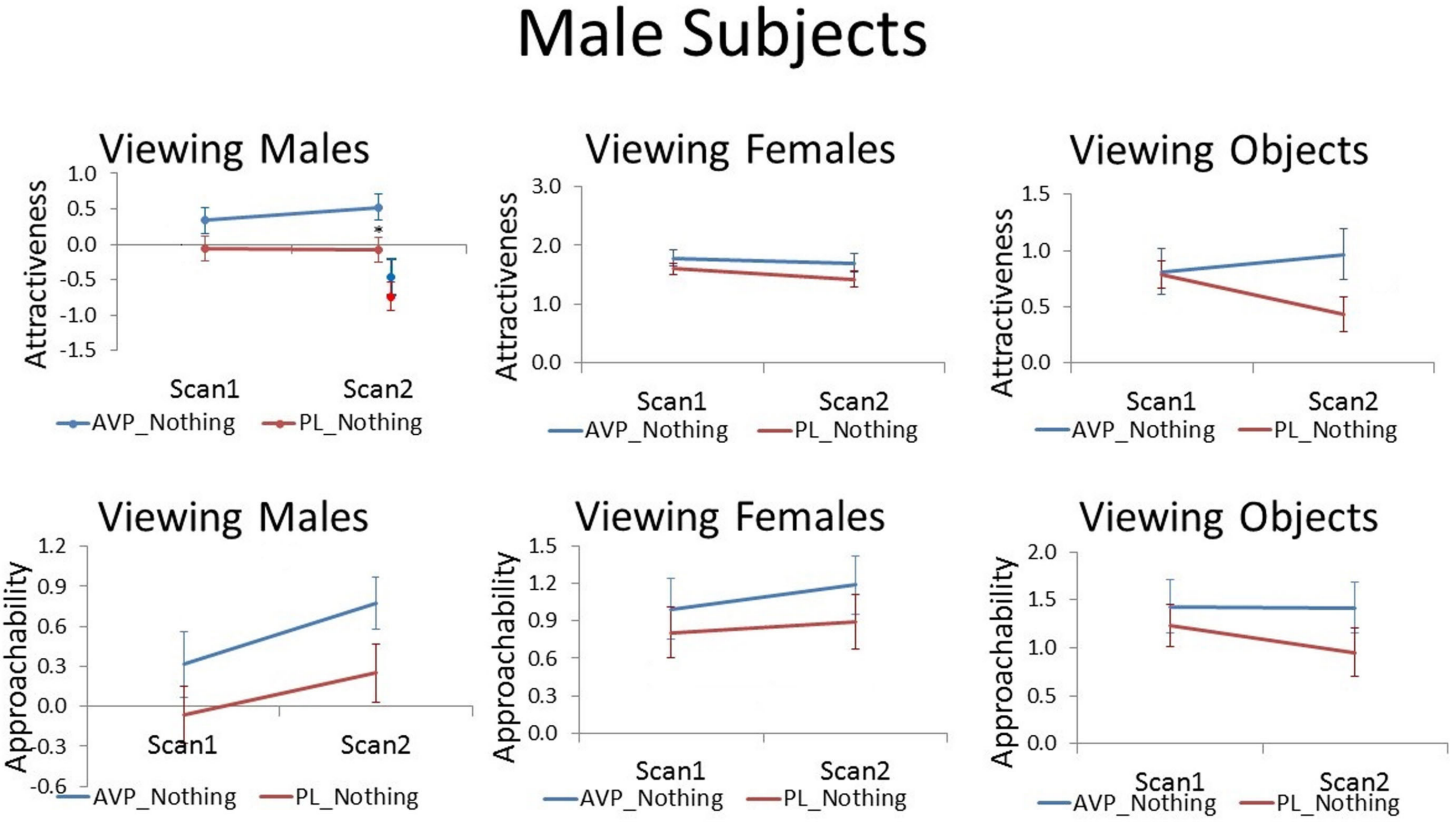
Subjective ratings of same-sex faces, other-sex faces, and objects in men, as a function of arginine vasopressin (AVP) vs. PL treatment and first vs. second scan. Error bars = ± 1 SE. *p < 0.05 for AVP vs. PL at scan 2.

Although AVP did not increase attractiveness ratings of male faces at scan 1, it did significantly increased attractiveness ratings of male faces at scan 2 [*t*(38) = 2.28, *p* = 0.03]. In the PL group, the number of days between scan 1 and scan 2 was negatively correlated with scan 2 attractiveness ratings of familiar male faces (*r* = −0.51, *p* = 0.02). That is, men rated male faces they had seen previously as less attractive as the scan interval increased. On the other hand, in the AVP group, there was no correlation between scan interval and scan 2 attractiveness ratings of familiar male faces (*r* = −0.15, *p* = 0.54). AVP effects appear to become more pronounced with longer scan intervals (Figure S1 in Supplementary Material). To determine if these marginal carryover effects generalized to the novel faces seen at scan 2, we also compared attractiveness ratings for novel male faces between the AVP and PL groups and found no significant difference [*t*(38) = 0.90, *p* = 0.38]. However, the effect of AVP did not significantly differ for familiar and novel faces [*F*(1, 38) = 1.14, *p* = 0.29] (Figure 2). AVP had no significant effect on approachability ratings of male faces at either scan 1 or scan 2. There was also no effect of AVP treatment on either attractiveness or approachability ratings of female faces at either scan 1 or scan 2. Finally, there was no significant effect of AVP treatment on either attractiveness or approachability ratings of objects, at either scan 1 or scan 2 (Table S1 in Supplementary Material).

### Neuroimaging Data

#### Whole Brain Analyses

In whole brain analyses, there was no effect of AVP treatment vs. PL treatment on the BOLD response to either same or other-sex faces in either men or women, at either scan 1 or scan 2.

#### ROI Analyses

##### Female Participants

**Figure 3:**
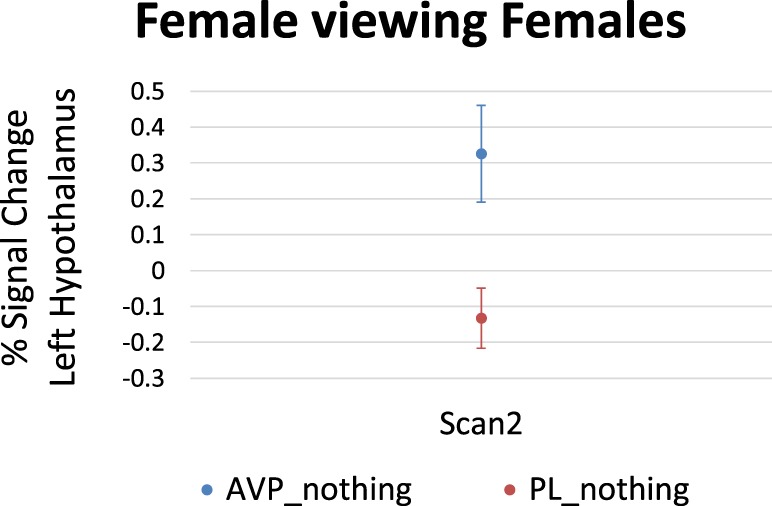
Arginine vasopressin (AVP) effects on lateral hypothalamic activation in females viewing female faces at scan 2. Error bars = ± 1 SE.

For women viewing female faces, there was no effect of AVP treatment at scan 1. On scan 2, AVP increased the response to female faces in the left hypothalamus (4 voxels, peak activation at MNI coordinate = −2, −4, −16; peak *z* = 2.68, *p* = 0.004) (Figure 3).

**Figure 4:**
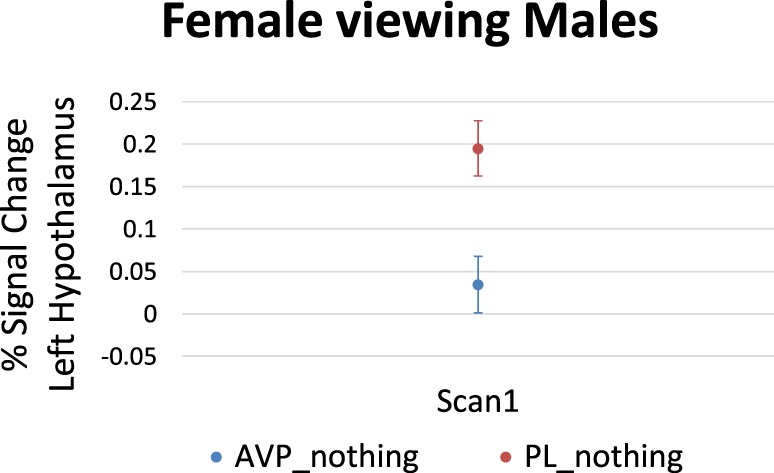
Arginine vasopressin (AVP) effects on lateral hypothalamic activation in females viewing male faces at scan 1. Error bars = ± 1 SE.

For women viewing male faces, AVP treatment decreased the left hypothalamus response at scan 1 (6 voxels, peak activation at MNI coordinate = −4, −4, −8; peak *z* = 2.65, *p* = 0.004) (Figure 4). There was no effect of AVP treatment on scan 2.

##### Male Participants

**Figure 5:**
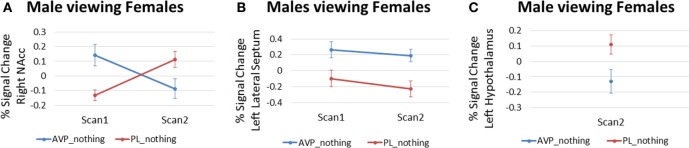
Arginine vasopressin (AVP) effects in males viewing female faces for **(A)** right nucleus accumbens, **(B)** left lateral septum, and **(C)** left hypothalamus. Error bars = ± 1 SE.

For men viewing female faces, AVP treatment on scan 1 increased the right NAc response compared with PL (3 voxels, peak activation at MNI coordinate = 6, 14, −8; peak *z* = 2.94, *p* = 0.002) (Figure 5A). However, the opposite effect was observed at scan 2, when AVP decreased both the right (23 voxels, peak activation at MNI coordinate = 8, 10, −6; peak *z* = −2.74, *p* = 0.003) (Figure 5A) and left NAc response to female faces (4 voxels, peak activation at MNI coordinate = −6, 2, −8; peak *z* = −2.88, *p* = 0.002). In addition to these effects within NAc, AVP treatment at scan 1 also increased the bilateral lateral septum response compared with PL (left septum = 6 voxels, peak activation at MNI coordinate = −2, −2, 12; peak *z* = 2.67, *p* = 0.004) (right septum = 3 voxels, peak activation at MNI coordinate = 4, −2, 14; peak *z* = 2.58, *p* = 0.005) (Figure 5B). AVP also increased the left, but not right, lateral septum response to female faces at scan 2 (3 voxels, peak activation at MNI coordinate = −2, 2, 10; peak *z* = 2.59, *p* = 0.005) (Figure 5B). While AVP had no effect on activation in the hypothalamus at scan 1, AVP decreased the left hypothalamus response to female faces at scan 2 (3 voxels, peak activation at MNI coordinate = −4, 2, −8; peak *z* = 2.78, *p* = 0.003) (Figure 5C). Finally, AVP had no effect on activation in the amygdala at either scan 1 or scan 2. While we could not accurately define a ventral pallidum ROI given its very small size and the lack of anatomical landmarks to guide its definition in the anatomical MRI, inspection of the uncorrected (*p* < 0.05) whole brain results showed a lack of AVP-related activation in the vicinity of the ventral pallidum.

For men viewing male faces, there was no effect of AVP treatment at either scan 1 or scan2.

## Discussion

Here, we show that administration of 40 IU intranasal AVP approximately 30 min before viewing faces influences both neural responses to and subjective ratings of those faces. In women, AVP treatment on the first scan day increased attractiveness ratings of female faces, and there was a trend for this effect to persist until scan 2 when no treatment was administered. AVP also increased the left hypothalamus response to female faces, but only on scan 2. Additionally, AVP decreased the left hypothalamus response to male faces on scan 1 only. In men, despite no effect on subjective ratings at scan 1, AVP increased attractiveness ratings of male faces at scan 2. AVP also increased the right NAc and bilateral lateral septum responses to female faces on scan 1. These effects persisted to scan 2 for the left lateral septum, but reversed for right NAc. Finally, AVP decreased activation to female faces within the left hypothalamus, but only for scan 2. Although these patterns are complex, three important points emerge. First, intranasal AVP can promote positive responses to same-sex faces in women and men, though on different time scales. Second, there is not always congruence between behavioral effects and influences in brain areas with functions presumably related to the behavioral responses; AVP increased attractiveness ratings in women on scan 1, but not activation in NAc, in which activity is correlated with positive assessments (46). Conversely, in men, AVP selectively increased activation in response to female faces in several areas, but did not affect behavioral responses. These patterns suggest either that some behavioral outputs may depend more on complex network or emergent processes than we currently capture, that areas in which activity was influenced are unrelated to the behaviors measured, and/or that our statistical methods are too conservative to capture such associations. Third, a single dose of intranasal AVP has the potential to induce long-lasting effects on behavioral and neural responses.

In a previous study, 20 IU intranasal AVP increased approachability ratings of women viewing female faces with neutral expressions (23). While we did not strictly replicate this effect, AVP-treated women did rate female faces as more attractive compared with PL-treated women. We believe attractiveness ratings in women are a generalized assessment of same-sex individuals and not a specific measure of sexual or romantic interest because heterosexual women in this and in our parallel studies rated the faces of other women as more attractive than the faces of men. Together, the two studies indicate that AVP selectively promotes positive assessments of the faces of other women in women. We did not measure the menstrual cycle phase of women in this study, and so cannot say whether AVP effects in women are moderated by cyclic hormones. In contrast to the effects of lower doses, which decreased social assessment of other men in our previous study, the current findings indicate that higher doses can, over time (see further discussion below), increase positive assessments of other men. Thus, different doses may produce divergent behavioral responses in men, a possibility also supported by dose differences found in our parallel study (under review, this issue). That AVP selectively enhanced assessments of same-sex faces in men and women is consistent with AVP/AVT’s effects in other species being dependent on the sex of stimulus (35, 36).

Arginine vasopressin’s ability to acutely increase assessments of other women in women on scan 1 or of other men in men on scan 2 was not associated with detectable alterations of brain activity, suggesting that those behavioral effects depend on modulation in areas outside the ROIs we examined, or on complex alterations in patterned activity that we did not measure. We had expected that AVP treatments that increase positive social assessments would also increase the ventral striatum response, which has been associated with increased positive ratings of other individuals (46). However, this was not the case. While these results are unexpected, NAc need not be tracking positive ratings in relation to the rewarding aspect of the faces. Indeed, if increased ratings of other female faces are part of a “tend and befriend” stress responses strategy, particularly in women (47), then AVP’s behavioral influences may not be directly related to reward and/or positive affect, but rather to stress/anxiety reduction. On the other hand, there was a parallel effect on behavioral and neural responses on scan 2 in women; AVP delivered prior to scan 1 marginally increased attractiveness ratings on scan 2 and increased responses to female faces in the lateral hypothalamus. AVP enhances aggression *via* actions in the hypothalamus in male hamsters but decreases aggression in female hamsters (48), though it is not yet known if AVP’s ability to inhibit aggression in females depends on activation or inhibition of hypothalamic activity. We did not measure responses directly associated with aggression, though increased social assessments of other women might be associated with decreased aggressive responses toward them. However, it is unclear why associations between increased hypothalamic activation on scan 2 would be related to increased attractiveness ratings in the absence of similar linkages on scan 1, or of linkages between the decreased activation in hypothalamic and behavioral responses to male faces on scan 1, unless AVP’s acute and lasting influences on behavioral and brain responses are associated with different mechanisms (see further discussion below). It is, therefore, also possible that influences on hypothalamic responses are completely unrelated to social assessments. Perhaps most important is simply that AVP did influence hypothalamic responses to faces, as AVP influences on hypothalamic responses to social stimuli, which mediate a variety of social behaviors in other vertebrates, have not previously been reported in humans.

In previously unpaired male prairie voles, AVP elevations in the ventral striatum that are concurrent with social contact with a novel female facilitate affiliative responses toward that individual. AVP appears to link the reward from mating with the identity of a particular female, resulting in a preference to associate with that female over others (49). Despite the independent evolution of pair bonding in prairie voles and humans and associated differences in life histories associated with pairing, males in both species are capable of forming such bonds in reproductive contexts, and AVP has been indirectly implicated in that process in human males (37). Although the design of this experiment was not identical to those used to test AVP’s role in pair bonding in prairie voles, in part due to the limitations associated with manipulating human subjects, we did presumably elevate AVP in single men while they were exposed to novel female faces. We recognized that the induction of social preferences related to pair bond formation in prairie voles requires continuous AVP administration during sustained social contact with a novel female (4, 36), so we did not expect our more limited manipulation to induce selective social attachments to the briefly presented faces. Nonetheless, we predicted that intranasal AVP might, particularly in light of the sustained elevations of the peptide in the brain that follow intranasal delivery (17) (discussed further below), enhance ratings indicative of increased attractiveness toward or tendencies to interact/affiliate with novel females while decreasing similar assessments of other males in relation to potential mate competition/guarding functions. We also predicted elevations of AVP during those limited “interactions” might increase activity in areas of the brain in which AVP promotes affiliative responses related to pair bonding with females and aggression toward males in voles if convergent peptide mechanisms associated with the promotion of emotional attachments in reproductive contexts evolved in the two species. These predictions were only partially supported. Although AVP did specifically augment bilateral responses in the lateral septum and ventral striatum to female faces, the responses in the ventral striatum were in the NAc, not the ventral pallidum, where AVP promotes affiliative responses relating to pairing in male prairie voles, and it did not augment ratings of female faces in this or our parallel study, in which two doses of AVP were administered, and in which men were only exposed to a single female face, but had more exposure to that face. Nor did the dose used in this study decrease responses to other males, but rather enhanced them over time. It is possible that our rating responses do not reflect responses related to affiliative and aggressive processes associated with pair-bonding/mate-guarding, or that our tests did not stimulate sufficient, concurrent dopamine release, which is also involved in stimulating partner preferences in male prairie voles (49). Indeed, the female faces were not even smiling and thus unlikely to have represented a potential romantic interest/partner. Of course, we also must acknowledge the possibility that AVP does not, as it does in male prairie voles, facilitate affiliative processes related to pair bonding in human males, and if it does, that it does so through different neural mechanisms, i.e., through activations in the lateral septum and NAc, rather than the ventral pallidum, and perhaps also through long-term downregulation of lateral hypothalamic responses to familiar females, which were lower only on scan 2. Indeed, comparative studies have suggested that common AVT/AVP mechanisms do not underlie pair bond formation in reproductive contexts in species in which such tendencies have independently evolved (50–52).

The reversal of AVP effects on male NAc activation to female faces at scan 2 was unexpected. However, this result may be consistent with another fMRI study showing that intranasal AVP effects differ for familiar and unfamiliar faces (27), as the same female faces were used on scan 1 and scan 2 in our study. Figure 5B shows an increase in NAc activation from scan 1 to scan 2 in the PL group. This might reflect increased reward from the female face over time as familiarity is established. It is possible that AVP accelerates this process so that NAc activity is already augmented at scan 1 in men who received AVP. The subsequent decrease in NAc activation from scan 1 to scan 2 could reflect a social habituation effect of AVP as has been reported previously for the closely related nonapeptide OT (53).

Arginine vasopressin modulation of lateral septum and NAc activation was specific to men, and AVP had opposing effects on hypothalamic activation in men and women viewing female faces. These results are consistent with accumulating evidence for sex differences in AVP effects in both humans and other animals (43, 44). In rats, for example, treatment with a V1aR antagonist in the lateral septum significantly increased social play in males while decreasing social play in females (54). In hamsters, hypothalamic AVP injection stimulated aggression in males, while inhibiting aggression in females (48). In humans, intranasal AVP augmented bilateral insula activation to positive social interactions in men, while having the opposite effect in women (26). Finally, with a face viewing paradigm similar to that used here, we previously showed that lower doses of AVP induced agonistic facial motor patterns and decreased perception of friendliness to same-sex faces in men, while inducing affiliative facial motor patterns and increased perceptions of friendliness to same-sex faces in women (23). These sex differences in AVP effects are accompanied by and may be attributable to widespread sex differences in V1aR distribution (44). Describing sex differences in human V1aR is an important task for future research.

No effects were observed in either whole brain analyses or in ROI analyses focused on the amygdala. The amygdala plays a critical role in threat detection (55–57), and AVP has been shown to modulate amygdala response in various contexts in both humans and non-humans (25, 58–60). Given that 20 IU intranasal AVP was previously shown to stimulate agonistic facial motor patterns and to increase skin conductance responses to same-sex faces in men (23), we expected AVP to increase amygdala activation to same-sex faces in men; however, this was not observed. The current study used 40 IU intranasal AVP. It is possible that the hypothesized effects on amygdala activation in face processing contexts would emerge at the 20 IU dose, which our parallel study found yields a more negative response than the higher dose.

Arginine vasopressin did not increase the subgenual ACC response to faces as reported previously (28). Although the study by Zink et al. and our study employed the same dose of AVP (40 IU), our study included only neutral rather than emotional faces, perhaps requiring less emotion regulation. In fact, work in other vertebrates has shown that testing contexts do influence the types of effects the peptide has (61). This seems to be true also in humans. A previous neuroimaging study showed that intranasal AVP effects on face-processing activity differed as a function of the familiarity of the face stimuli. Specifically, AVP effects were found for unfamiliar, but not familiar, faces (27). Our stimuli consisted of nine photographs of two male and two female models (for scan 1). Thus, subjects were presented with highly similar stimuli with which they were quickly familiarized, because we wanted to mimic the increased familiarity that would typically occur during an ongoing social interaction. However, it is possible that the familiarity of our stimuli may have dampened some AVP effects. Finally, we began scanning at 32 min post-AVP administration as compared with 56 min for Zink et al. These variables might help to explain the discrepant results.

There was evidence that some AVP effects persisted until the second scan day when no treatment was given. Attractiveness ratings of other female faces remained marginally higher in women given AVP on the first day than in women given PL on the first day, and responses to female faces in the left lateral septum in men were similarly increased by AVP on both scans. Additionally, some effects only appeared on scan 2, including increased attractiveness ratings of other men in men and decreased activation in the lateral hypothalamus in response to female faces, or reversed across days, most notably the increased activation in the NAc in response to female faces in men on scan 1, but decreased activation on scan 2. Thus, AVP appears to have effects on face processing that likely persist beyond the presence of drug in the system, as AVP’s half-life in brains is less than 1 min, though the rate decreases over time (62) and its peptide fragment’s, which can have behavioral effects, is 6.5 h in tissue (63). The exogenous drug should have thus been cleared by the second scan. To some extent, our predication that AVP would produce selective, long-lasting effects on responses to previously viewed faces when AVP levels were elevated was supported in the behavioral data, in that responses to novel faces on scan two were not elevated. AVP can enhance social recognition memory in rodents (64, 65), in some cases, through the activation of OT receptors (66, 67), and intranasal AVP has been shown to enhance the encoding of happy and angry faces in men (21). AVP influences on the encoding of the faces on the first trial could, therefore, have altered their perception on the second trial, though it is unclear how acute influences on face processing may produce prolonged influences on subjective responses of the faces or on neural responses to those faces. Importantly, behavioral effects did not appear to lessen as a function of time since AVP administration, which suggests they could be long lasting. This is consistent with lasting effects associated with different doses of AVP in our parallel study (under review; this issue).

The mechanisms through which AVP may induce lasting or delayed effects on subjective responses to faces are unclear. AVP can enhance social recognition, but most such studies involve AVP administration after interactions with an individual, and the effects, to our knowledge, have not been observed more than 24 h after administration (68). If acute VP increased how familiar the faces seemed, it could have, to the extent that increased familiarity increases subjective ratings, led to sustained enhancements in those responses, potentially through synaptic remodeling, which AVP can promote (69–71). Although it seems unlikely that a single administration of AVP would be sufficient to induce such alterations, it should be kept in mind that, despite AVP’s short half-life in tissue, the elevations observed in the brain after intranasal delivery were still apparent in the original studies by Born et al. (17), with no signs of decreasing, 80 min after delivery, suggesting that intranasal AVP might, perhaps through feed-forward mechanism (72), trigger large and sustained elevations of AVP within the brain that could induce such changes.

We cannot draw any conclusions about the types of receptors that mediate these effects. It is possible that higher doses of AVP produce positive social assessments in women through the activation of related oxytocin receptors, though it should be noted that the positive effects in women, at least, are consistent with those observed for lower doses that also increased anxiety, which is not consistent with oxytocin receptor activation (23). Even if the effects observed here are, at least in part, the results of receptor cross talk, this would not negate the clinical relevance of these peptides when considering their use as therapeutics nor the potential importance of such mechanisms for normative social functioning. Some of the AVP’s behavioral effects depend on activation of the OT receptor (66, 67), and in some cases, AVP’s effects may depend on the simultaneous activation of both AVP and OT receptors (73). Clearly, we need to learn more about the local concentrations of peptides released within local circuits in different social contexts relative to the amounts that reach those areas through intranasal delivery. It will also be interesting to determine if different patterns of receptor activation, perhaps as a function of the amounts of peptide released endogenously or the dose applied exogenously, may produce different behavioral outcomes. Given evidence that OT and AVP receptors can heterodimerize (74), it is even possible some behavioral effects depend on interactions with complex combinations of membrane receptor proteins.

In summary, we show that treatment with 40 IU intranasal AVP increases positive ratings (attractiveness) of same-sex faces in women and that these effects may persist for several days. AVP also increased attractiveness ratings of male faces in men at scan 2 only. fMRI data show that AVP provoked acute increases in right NAc and bilateral lateral septum responses to female faces among men, with the left lateral septum response persisting until scan 2 while the right NAC response reversed at scan 2. AVP also modulated the left hypothalamus response to faces in both men and women, in some cases only on scan2. Work is ongoing to determine if AVP effects within these data sets are modulated by individual variables such as genotype, personality, or attachment style as previously reported for both vasopressin and the closely related oxytocin (75–78).

## Ethics Statement

All subjects gave written informed consent, and the study was approved by the Emory University Institutional Review Board and the U.S. Food and Drug Administration.

## Author Contributions

JR designed the study, supervised analysis, interpreted data, and wrote the paper. TL analyzed data and edited the paper. XC analyzed data and edited the paper. PG collected and analyzed data and edited the paper. EH served as study physician and edited the paper. RT designed the study, interpreted data, and wrote the paper.

## Conflict of Interest Statement

The authors declare that the research was conducted in the absence of any commercial or financial relationships that could be construed as a potential conflict of interest. The reviewer, EA, and handling editor declared their shared affiliation, and the handling editor states that the process nevertheless met the standards of a fair and objective review.

## Footnotes

^1^http://www.randomizer.org.

^2^http://www.fmrib.ox.ac.uk/fsl/.

^3^https://fsl.fmrib.ox.ac.uk/fsl/fslwiki.

## Supplementary Material

Figure S1 Anatomically defined regions of interest in **(A)** nucleus accumbens, **(B)** amygdala, **(C)** hypothalamus, and **(D)** lateral septum.

Figure S2 Scan 2 attractiveness ratings in the arginine vasopressin (AVP) and placebo (PL) groups as a function of scan interval for female (left) and male (right) participants.
