# Erratum: Arginine Vasopressin Effects on Subjective Judgments and Neural Responses to Same and Other-Sex Faces in Men and Women

**DOI:** 10.3389/fendo.2017.00322

**Published:** 2017-11-16

**Authors:** Office Frontiers Production

**Affiliations:** ^1^Frontiers Media SA, Lausanne, Switzerland

**Keywords:** vasopressin, fMRI, face processing, sex differences, nucleus accumbens

In the original article, older versions of Figures 1 and 2 were used.

**Figure 1:**
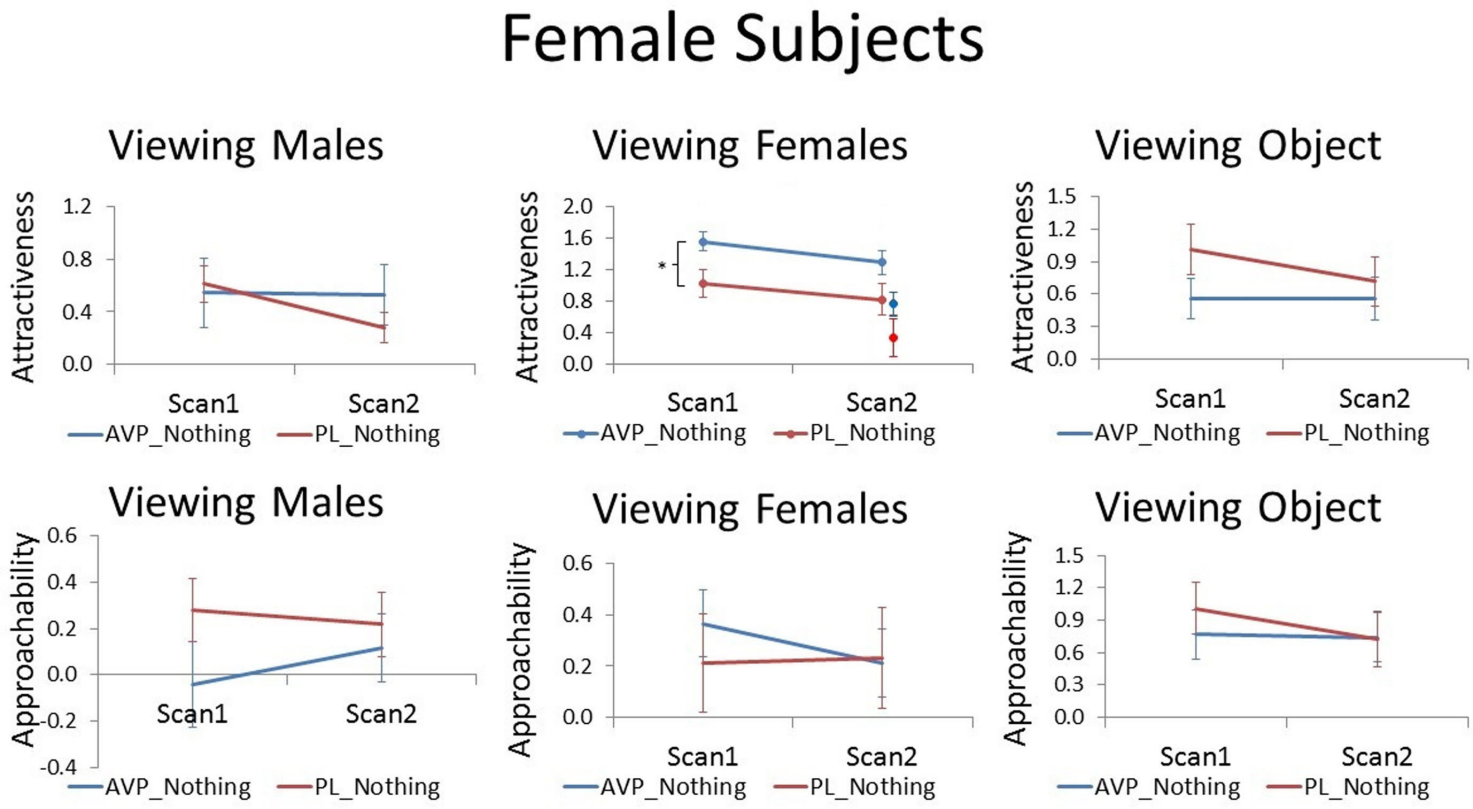
Subjective ratings of same-sex faces, other-sex faces, and objects in women, as a function of arginine vasopressin (AVP) vs. PL treatment and first vs. second scan. For attractiveness ratings of female faces, data for novel faces at scan 2 are plotted to the right of familiar faces. Error bars = ±1 SE. ^*^p < 0.05 for AVP vs. PL at scan 1.

**Figure 2:**
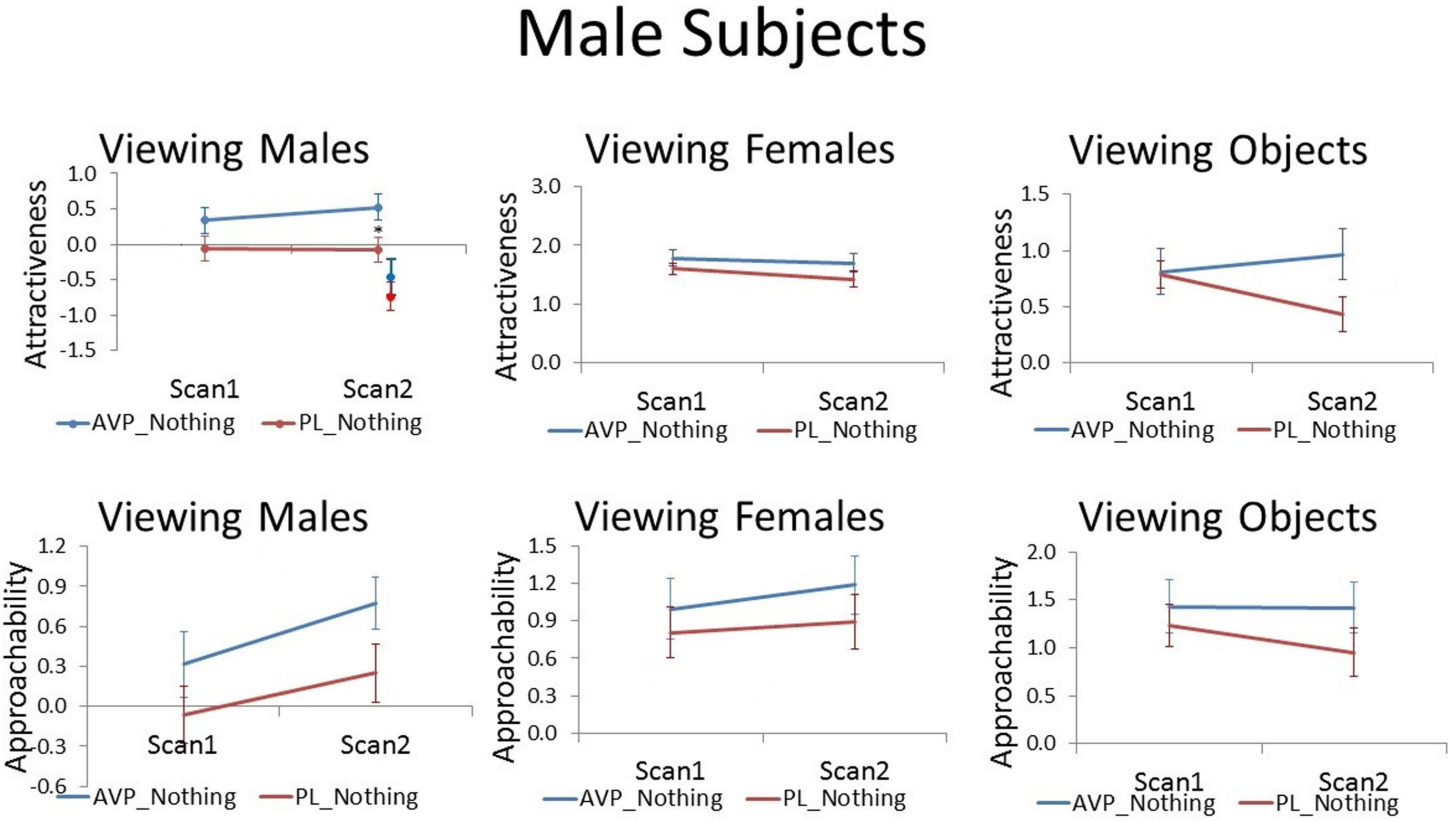
Subjective ratings of same-sex faces, other-sex faces, and objects in men, as a function of arginine vasopressin (AVP) vs. PL treatment and first vs. second scan. Error bars = ±1 SE. ^*^p < 0.05 for AVP vs. PL at scan 2.

The correct version of the Figures appears below. This error does not change the scientific conclusions of the article in any way.

The publisher apologizes for this mistake and the original article has been updated.

## Conflict of Interest Statement

The authors declare that the research was conducted in the absence of any commercial or financial relationships that could be construed as a potential conflict of interest.

