# Supplementary figures and images for: Arginine Vasopressin Effects on Subjective Judgments and Neural Responses to Same and Other-Sex Faces in Men and Women
Source: Front Endocrinol (Lausanne). 2017 Aug 21;8:200. doi: 10.3389/fendo.2017.00200 (PMC5566575; doi:10.3389/fendo.2017.00200)

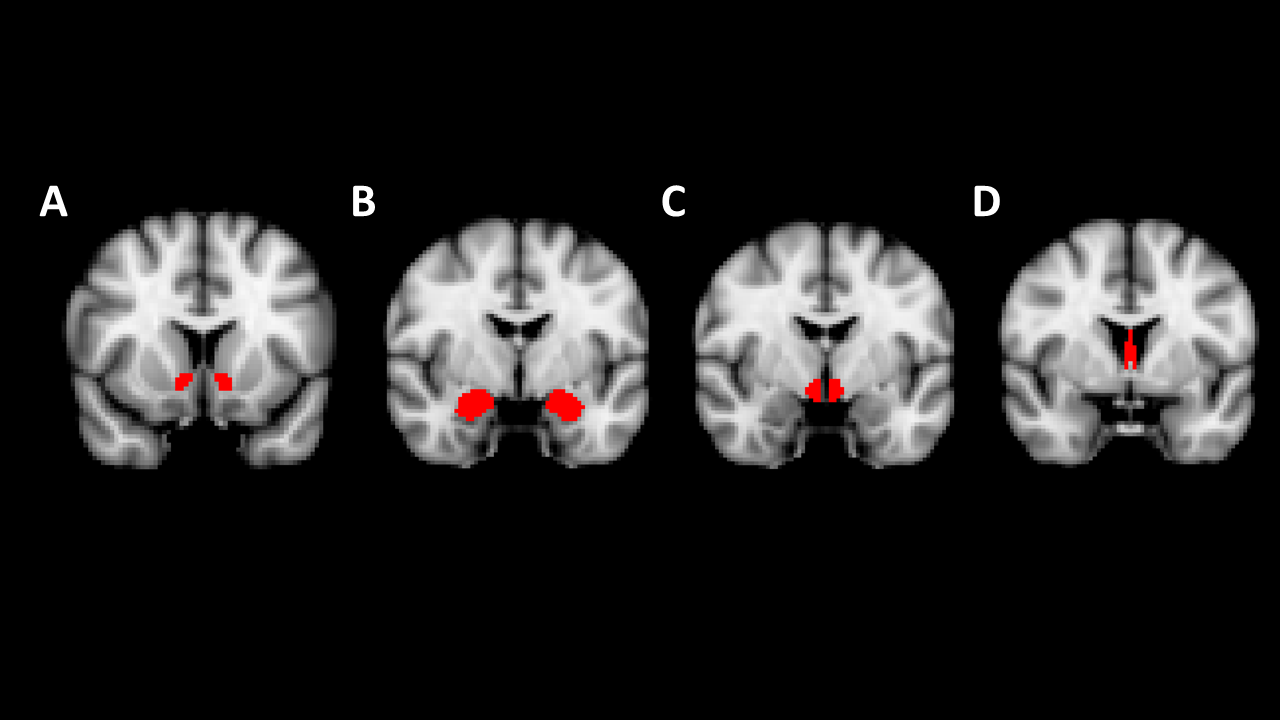

Supplement: Figure S1 — Anatomically defined regions of interest in (A) nucleus accumbens, (B) amygdala, (C) hypothalamus, and (D) lateral septum. [file image_1.tif]

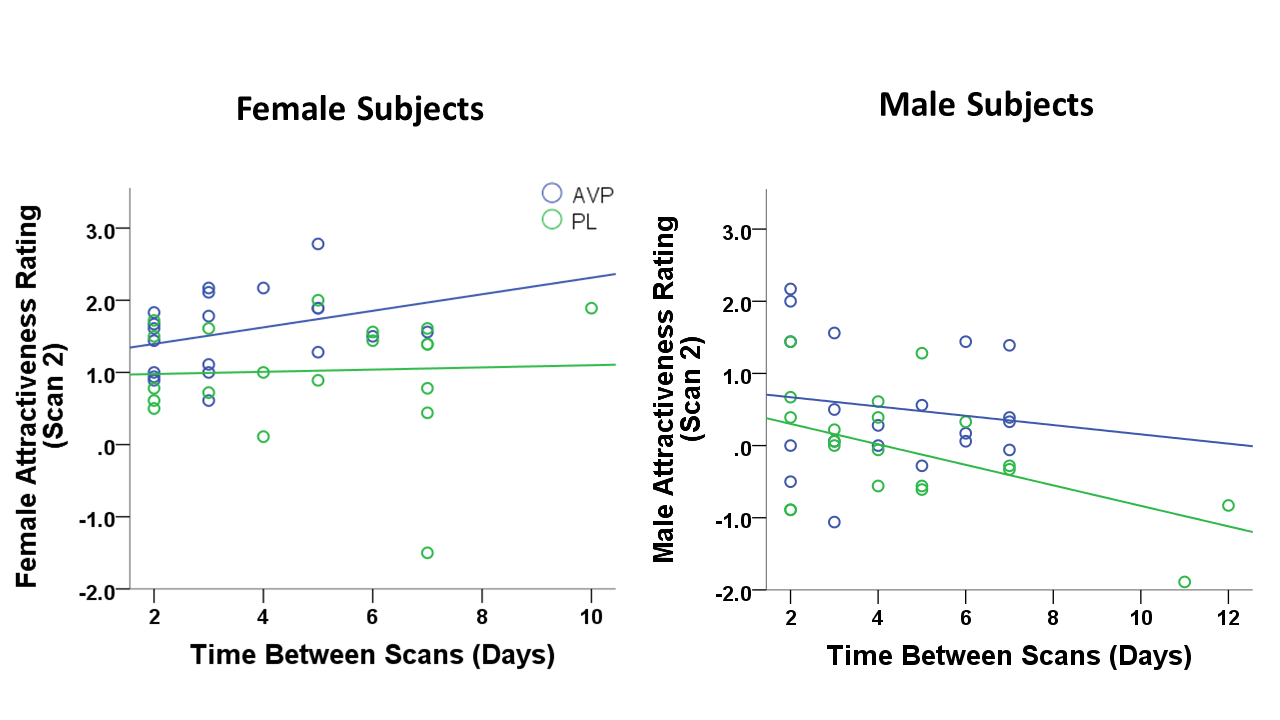

Supplement: Figure S2 — Scan 2 attractiveness ratings in the arginine vasopressin (AVP) and placebo (PL) groups as a function of scan interval for female (left) and male (right) participants. [file image_2.tif]
